# Secondary Prevention of Dementia: Combining Risk Factors and Scalable Screening Technology

**DOI:** 10.3389/fneur.2021.772836

**Published:** 2021-11-15

**Authors:** Triin Ojakäär, Ivan Koychev

**Affiliations:** ^1^Sharp Therapeutics Ltd., London, United Kingdom; ^2^Department of Psychiatry, University of Oxford, Oxford, United Kingdom

**Keywords:** dementia, Alzheimer's disease, secondary prevention, diabetes, hypertension, chronic kidney disease, digital technology, blood-based biomarkers

## Abstract

Alzheimer's disease (AD) is a progressive neurodegenerative disorder that is the most common cause of dementia. Over a third of dementia cases are estimated to be due to potentially modifiable risk factors, thus offering opportunities for both identification of those most likely to be in early disease as well as secondary prevention. Diabetes, hypertension and chronic kidney failure have all been linked to increased risk for AD and dementia and through their high prevalence are particularly apt targets for initiatives to reduce burden of AD. This can take place through targeted interventions of cardiovascular risk factors (shown to improve cognitive outcomes) or novel disease modifying treatments in people with confirmed AD pathology. The success of this approach to secondary prevention depends on the availability of inexpensive and scalable methods for detecting preclinical and prodromal dementia states. Developments in blood-based biomarkers for Alzheimer's disease are rapidly becoming a viable such method for monitoring large at-risk groups. In addition, digital technologies for remote monitoring of cognitive and behavioral changes can add clinically relevant data to further improve personalisation of prevention strategies. This review sets the scene for this approach to secondary care of dementia through a review of the evidence for cardiovascular risk factors (diabetes, hypertension and chronic kidney disease) as major risk factors for AD. We then summarize the developments in blood-based and cognitive biomarkers that allow the detection of pathological states at the earliest possible stage. We propose that at-risk cohorts should be created based on the interaction between cardiovascular and constitutional risk factors. These cohorts can then be monitored effectively using a combination of blood-based biomarkers and digital technologies. We argue that this strategy allows for both risk factor reduction-based prevention programmes as well as for optimisation of any benefits offered by current and future disease modifying treatment through rapid identification of individuals most likely to benefit from them.

## Introduction

Alzheimer's disease (AD) is a progressive neurodegenerative disorder that is the most common cause of dementia accounting for 60–80% of all dementia cases (1). In the UK, the projected prevalence rate for dementia for individuals aged 65 and over has reported to be 7.2% (2). This constitutes 1 in every 14 individuals over the age of 65. Worldwide, it has been estimated that 46.8 million people are living with dementia, with the prevalence rates nearly doubling every 20 years (1). Continuous advances in healthcare have increased life expectancy, but as a result the number of individuals suffering from age-related diseases such as AD is on the rise. 2021 was a watershed moment for the field with the approval of the first, ostensibly, disease modifying treatment—an amyloid targeting therapy (aducanumab). This has given further impetus to the need to identify AD pathology with a focus on detecting disease in its earliest possible stages. Decades of research into the constitutional and environmental risk factors of AD have revealed the complexity of its pathology, but also highlighted the significant contribution of modifiable risk factors. It has been estimated that ~40% of AD cases worldwide could be attributable to 12 potentially modifiable risk factors (3). Controlling for these risk factors could prevent up to 1–3 million cases globally (4).

Cardiovascular risk factors such as hypertension and diabetes have been identified amongst the 12 potentially modifiable risk factors for dementia in the 2020 Lancet Commission on dementia prevention (3). Hypertension is estimated to carry a relative risk of 1.6 (95% CI 1.2–2.2) but interventional studies have shown that controlling it through therapy reduces the risk by ~10% (5). Diabetes, which also affects the cardiovascular system has been identified among the major risk factors for dementia with relative risk of 1.5 (95% CI 1.2–1.8) (3). The effect of diabetic control on the risk of dementia is less clear with studies reporting mixed results. A meta-analysis of cohort studies has reported that individuals taking metformin were less likely to develop cognitive impairment compared to those taking other medications or no treatment at all (6). However, other studies have reported no benefits of diabetes control on cognitive health (7). Hypertension and diabetes are known to cause multi-organ damage and have been identified as the primary causes of chronic kidney disease (CKD) (8, 9) which in turn has been associated with risk for cognitive decline (10). Taken together, diabetes, hypertension and CKD are inter-linked cardiovascular disorders that can help identify those most at risk for cognitive decline.

Prospective studies of at-risk populations have revealed that AD pathology is present decades before a clinical diagnosis is made (11, 12). The long pre-clinical phase offers a window of opportunity for secondary prevention of dementia through risk factor control and/or aetiological treatment. The amyloid hypothesis of AD argues that amyloid-β (Aβ) plaque accumulation is the initiating event, triggering a cascade of tau protein hyperphosphorylation (creating neurotoxic neurofibrillary tangles), synaptic loss, neurodegeneration and eventually cognitive decline (13). Crucially, when clinical symptoms of cognitive impairment appear, the underlying AD pathology has already entered its advanced stage, arguably limiting the impact of any interventions attempted at that phase (14). The long pre-clinical phase of AD provides the opportunity for detecting underlying pathology before clinical symptoms appear (15). Diagnosing the biological state of AD has accordingly become a major focus of the research field.

Incremental improvements in the cerebrospinal fluid (CSF) and positron emission tomography (PET) methodology have provided the means to detect early signs of amyloid and tau pathology in the living patient. However, the utility of these methods to detect AD at scale is precluded by their invasiveness, expensiveness and dependence on expertise and technology typically confined to major academic centers. Thus, to take full advantage of the opportunity offered by the protracted preclinical and prodromal dementia disease stages, more appropriate tools are needed to monitor at-risk groups. Recent advancements are proving new opportunities for this through blood-based biomarkers and digital technologies. Blood-based biomarkers have significant advantage over CSF and PET methods due to their time and cost-efficiency, reduced invasiveness and infrastructure availability to support large scale testing. There is substantial evidence that shows the utility of blood-based biomarkers in predicting dementia progression (16), conversion from Mild Cognitive Impairment (MCI) to AD (17) and detecting risk for future AD in healthy aging adults (18). Further data is accumulating on their usefulness in distinguishing different dementia-causing pathologies (19). Concurrently there has been significant investment in the development of digital biomarkers for monitoring cognitive, sensory and motor changes in individuals at risk of AD. Sensory and motor changes can predict AD onset 10–15 years before clinical symptoms appear (20, 21) making them an important complement to fluid biomarkers. The deep societal penetration of digital device use across age strata offers an until now unavailable opportunity for individuals to monitor their risk of AD without having to visit a specialized clinic. Active monitoring devices allow users to measure their cognitive abilities through digital assessments that target specific metrics previously been associated with AD (22). In contrast, passive monitoring devices, record users' activity and engagement with their smart device without having to perform any explicit tasks. For instance, the typing speed and number of pauses during typing on a smartphone can discriminate between individuals with cognitive impairment and healthy controls (23). Therefore, between the sensitivity of blood biomarker assays and the low implementation cost of digital technologies, a realistic opportunity has emerged to conduct secondary prevention programmes.

The purpose of this review is to make the case for the secondary prevention of dementia through (i) defining the chronic physical conditions most appropriate for active AD monitoring through the strength of evidence and prevalence and (ii) to propose scalable and cost-effective tools for monitoring high-risk populations for dementia.

## Type II Diabetes

### Epidemiology

Diabetes mellitus (DM) is a chronic metabolic disease affecting ~463 million adults globally (24). With the numbers rising significantly, it is estimated that the global prevalence of diabetes will reach 548 million by 2045 (24). A growth rate this high makes diabetes mellitus one of the most significant health challenges of the 21st century, with type II diabetes mellitus (T2DM) being the fastest growing chronic disorder worldwide (25). Type 2 diabetes is characterized by persistent hyperglycaemia (26) and can be attributed to multifactorial integrating factors such as genetics (27), age, socioeconomic status, education (28), as well as, modifiable risk factors including diet (29), smoking (30), and levels of physical activity (31).

The effectiveness of treatment options for diabetes have improved incrementally over the past few decades (32), thus increasing the lifespan of patients. Despite this positive trend, a growing body of epidemiological research suggests that individuals with type II diabetes are at an increased risk of developing neurodegenerative diseases such as AD (33). The Rochester dementia incident cohort study (34) conducted between 1970 and 1984 was among the first to demonstrate a significant association between diabetes and cognitive decline. It found that individuals with diabetes have a significantly higher risk for AD than controls. Similar findings were demonstrated by the Rotterdam Study (35) where individuals with diabetes were at a 2-fold risk of Alzheimer's disease compared to controls. Similarly, in the Finnish Vantaa cohort Ahtiluoto et al. (36) reported that diabetes doubled the incidence of dementia, AD and vascular dementia, and increased mortality. In this post-mortem study, individuals with diabetes had lower levels of amyloid plaques and tangles relative to non-diabetics but were more likely to have cerebral infarcts. Individuals with diabetes were shown to be more prone to extensive vascular pathology, which independently or combined with AD-type pathology (especially in APOE e4 carriers) results in a higher risk for dementia. These findings have also been confirmed in a meta-analysis of cross-cultural studies looking at the association between T2DM and AD (37). It found that the AD risk for individuals with diabetes was significantly higher relative to those without diabetes. When looking at ethnic differences, the relative risk for Caucasian populations was slightly lower compared to Asian populations. In summary, there is consistent evidence that points to a link between T2DM and increased risk for cognitive decline and dementia, with risk being approximately double for those with a diabetes diagnosis. In terms of dementia etiology, T2DM appears to be more tightly associated with vascular than AD causes.

### Mechanism

There are many possible interacting mechanisms that contribute to the risk of dementia in individuals with T2DM. Firstly, T2DM associates with a pro-coagulation state which increases the risk for cerebrovascular events. Vascular risk factors and vascular events are an important determinant for brain atrophy and vascular brain lesions (38) that consequently increase the rate of cognitive decline (39). A systematic review of brain imaging studies has shown that T2DM is consistently associated with cerebral and lacunar infarcts (40) with the associated brain volume loss rate being equivalent to 3–5 years of healthy aging (41). Brain atrophy and brain lesions have been shown to accelerate in patients with DM even when cognitive differences are not detected (42). Furthermore, the presence of hypertension in diabetic patients has been associated with even greater cerebral atrophy than diabetes alone (43).

Chronic hyperglycaemia, which is central to T2D, is measured via glycosylated hemoglobin (HbA1c) and has been proposed as a direct mechanism linking T2D and cognitive decline. Research has shown that higher average glucose levels are associated with increased risk of dementia in both individuals with and without diabetes (44). Marden, Mayeda (45) reported that each percentage point increase in HbA1c was associated with a 0.052 unit decrease on a custom memory score per decade even in individuals without clinical diabetes. In a population-based cohort study HbA1c of 6.2% or greater predicted faster cognitive decline in individuals aged between 65 and 88 even after adjusting for age, sex, education, and APOE ε4 status (46). A cross-sectional study of patients with T2D showed a non-linear association between HbA1c levels and cognitive function that indicated a bell-shaped relationship with low and high HbA1c levels affecting cognitive decline (47). There is consistent evidence that links HbA1c levels with cognitive decline, but more research is needed to determine the temporal order of the effect.

Mechanisms for induction of AD-specific processes have also been proposed. Oxidative stress, mitochondrial dysfunction and chronic inflammation have been proposed to be key contributors (48). AD pathology is explained by the formation of neurofibrillary tangles and the build-up of extracellular β amyloid plaques, which both are facilitated by insulin resistance, the main characteristic of T2DM. Insulin resistance leads to a reduction in the activation of protein kinase B, a protein that holds an important role in glucose metabolism and the inhibition of glycogen synthase kinase 3 beta (GSK3β), a main kinase that phosphorylates the tau protein. An escalation in the activation of GSK3β may lead to the over-phosphorylation of the tau protein, which could explain the formulation of neurofibrillary tangles seen in individuals with AD (48). Furthermore, advanced glycation end products (AGE) which play a critical role in diabetes may increase in the brain due to hyperglycaemia and increase neuronal cell death (49).

Receptor for advanced glycation end products (RAGE) is a member of the immunoglobulin superfamily of cell surface molecules and has been proposed as an important link between T2DM and neurodegeneration. Research suggests that RAGE acts as an inflammatory intermediary and an inducer of oxidative stress leading to pathophysiological changes in the brain (50). An increase in oxidative stress with reduced antioxidant capacity can also lead to mitochondrial damage (51, 52). RAGE contributes to the production and accumulation of Aβ and neurofibrillary tangles, as well as overall neuronal degeneration. Furthermore, RAGE plays an important role in the pathogenesis of Aβ and increased tau phosphorylation, which are both associated with AD pathology (53).

Insulin resistance, a key contributor to diabetes, is also associated with inflammation (54). Systemic inflammation is thought to play a critical role in neurodegeneration and AD pathology. Systemic inflammation is characterized by release of pro-inflammatory cytokines and chemokines from the immune-related cells into the blood. These cytokines can lead to a pro-inflammatory environment in the central nervous system by entering the brain through the blood-brain barrier. Systemic inflammation can give rise to reactive, pro-inflammatory microglia and astrocytic phenotypes, that can also bring about tau hyperphosphorylation and Aβ amyloid oligomerization (55). A pro-inflammatory state has also been shown to directly contribute to the risk of coagulation and may additionally contribute to the risk for cerebrovascular events observed in T2DM (56).

### Interaction With AD Factors

Knowing the pathophysiological changes triggered by diabetes, it is important to distinguish individuals at highest risk of AD pathology for early prevention and treatment. Diabetic APOE ε4 carriers have a significantly higher risk for AD compared to individuals with either factor and individuals with both factors have demonstrated a higher number of hippocampal neuritic plaques and hippocampal and cortical neurofibrillary tangles, as well as, an increased risk for cerebral amyloid angiopathy (57). The interactive effect of type 2 diabetes and AD risk factors on the rate of functional decline has been investigated in cognitively healthy individuals through the Alzheimer's Disease Neuroimaging Initiative (58). The interaction between diabetes and AD features (cognitive decline APOE ε4 carriership, cerebrospinal fluid β-amyloid, total tau (t-tau) and hyperphosphorylated-tau (p-tau) showed that individuals with both diabetes and at least one AD feature had a faster functional decline rate than those without both factors. Of the individual AD features, subtle objective cognitive decline, APOE ε4 carriership, p-tau and tau but not CSF β-amyloid all accelerated functional decline. This study indicated that while diabetes likely accelerates AD pathology, it may be that it does so primarily through tau mediated mechanisms.

Finally, age and sex specific incident rates of AD in diabetic individuals show demographic differences: the risk is reportedly higher in diabetic women vs. diabetic men (59) and the risk is further exacerbated for older women (60).

### Effect of Treatments

Provided there are shared mechanisms involved in T2D and dementia, diabetes treatment could potentially provide an avenue to secondary prevention of cognitive decline. Results from interventional studies supporting this rationale have been mixed. Monotherapy with sulfonylurea has been found to decrease the risk of AD while combination therapy using non-sulfonylurea insulin secretagogue showed the opposite effect (59). However, after adjusting for underlying risk factors and duration of diabetes since diagnosis, neither monotherapy nor combination therapy with oral antidiabetic medications were associated with AD risk.

Rosiglitazone, an antidiabetic pharmacotherapy that optimizes endogenous insulin use and has systemic anti-inflammatory effects has also been shown to improve scores on cognitive measures after 6 months of treatment (61). Risner et al. (62) recruited mild to moderate AD patients to a randomized placebo-control trial and followed up for 24 weeks. There was a significant improvement in the cognitive assessments scores of APOE ε4 non-carriers who were given the rosiglitazone treatment compared to controls. APOE ε4 carriers not only did not improve cognitively but actually declined at the lowest treatment dose. These results suggest that the benefit of anti-diabetic medication may be limited in individuals at risk for AD-specific neurodegeneration.

Intranasal insulin has also been investigated as a potential protective treatment against further cognitive decline in those with memory impairments. Reger et al. (63) administered insulin treatment to individuals with memory-impairments and controls and found that the positive effects of treatment were stronger for memory impaired subjects who were APOE ε4 non-carriers compared to cognitively impaired non-carriers and cognitive healthy controls. Furthermore, APOE ε4 carriers showed poorer recall following intranasal insulin on one of the memory tests. These findings provide further support to the concept that diabetes agents may be more effective in those with non-AD pathology.

## Hypertension

### Epidemiology

Hypertension refers to systolic blood pressure above 140 mmHg and/or diastolic blood pressure above 90 mmHg which both increase with age (64). Hypertension is a major risk factor for cardiovascular disease as well as vascular dementia (65, 66). There is also evidence to suggest that hypertension increases the risk of AD-specific neurodegeneration. To understand AD prevalence in individuals with hypertension, Guan et al. (67) carried out a meta-analysis of longitudinal studies and found that out of 9 studies only one showed an association between hypertension and increased AD risk. Similarly, Power et al. (68) carried out a systematic review and meta-analysis to investigate the link between hypertension and AD. No clear relationship between hypertension and AD was found in the 18 studies analyzed. The lack of association in these studies have later been attributed to methodological flaws such as short follow-up times and lack of mid-life blood pressure measures. In a meta-analysis, Norton et al. (69) demonstrated that individuals with high mid-life blood pressure are at an increased risk for developing AD later in life. More recently, Walker et al. (70) examined the association of mid- to late-life blood pressure patterns with subsequent dementia, MCI and cognitive decline in prospective population-based cohort and showed that individuals combining mid-life hypertension and late-life hyper- or hypo-tension had significantly higher risk of dementia relative to those who maintained normal blood pressure levels during their adult years. In addition, systolic but not diastolic blood pressure shows a significant association with AD risk, while diastolic shows no association (71). These results have been interpreted as evidence that mid-life hypertension is the critical risk factor for dementia with late-life hypotension being a feature of prodromal dementia thus obfuscating the relationship between the two in older age.

### Mechanism

The link between dementia and hypertension is often explained by micro- and macrovascular complications arising through chronically elevated blood pressure. Accordingly, hypertension is one of the main risk factors for cerebrovascular events such as stroke, cerebral infarcts and the development of ischemic white matter lesions. While the more dramatic events constitute medical emergencies and are easy to detect, cerebral infarcts can also happen without focal symptoms making early detection and treatment difficult. Undiagnosed cerebrovascular disorders increase with age and can play an important role in the development of AD in individuals with hypertension. Untreated hypertension can predict the severity of neurofibrillary tangles and neuritic plaques in the brain (72) further suggesting a direct link between AD and hypertension. It has been proposed that hypertension aggravates Aβ- induced cerebromicrovascular impairments in AD and accelerates its progression. Tau pathology has also been linked to hypertension in a mouse model where experimentally induced hypertension worsened tau-related motor dysfunction (73). A study in patients demonstrated that after adjusting for Aβ levels in the CSF, lobar micro bleeds associated with high blood pressure are linked to faster cognitive decline and higher levels of p-tau in CSF samples (74). Moreover, in a mouse model it has been demonstrated that Aβ and tau may interact to compromise brain vascular function in AD (75). Thus, suggesting that tau may further aggravate microvascular Aβ deposition and its effects on AD.

Over time hypertension has been shown to cause hypertrophy and stiffening of arterial walls which associates with reduced blood flow in the microvasculature (76). This in turn affects the oxygen and nutrient supplies to tissues which may initiate or accelerate the AD pathophysiological cascade e.g., by impairing microglial function (77). Moreover, capillary loss behind the lack of cerebral blood flow can negatively affect the clearance of Aβ. Cerebral blood vessels play an important role in exchanging Aβ between blood and the brain, and therefore, changes in the cerebrovascular function can negatively affect its clearance from cortex (78). In support to the role of hypertension early in the AD process, reduced cerebral perfusion has been demonstrated in preclinical stages of AD (79). Furthermore, it has been hypothesized that hypertension leads to blood-brain barrier (BBB) breakdown by interactive mechanisms involved in inflammation, oxidative stress and vasoactive substances. The increased BBB permeability disrupts central nervous system homeostasis, exposing it to potentially cytotoxic factors such as inflammatory cytokines which has been argued to associate with accelerating neurodegeneration (80).

A close interactive relationship between hypertension and inflammation has also been proposed. The interaction between inflammation and hypertension may be more than additive leaving individuals with comorbidities at an increased risk for AD (81). In a mouse model, hypertension triggered hypoperfusion and neuroinflammation in both cortex and hippocampus. Inflammatory response was even higher as Aβ deposition became more detectable (82). Moreover, to understand the role of neuroinflammation in hypertension induced Aβ pathology, immune system activating and inhibiting treatments were compared. The former but not latter reduced amyloid load, indicating that controlling inflammation with immune system stimulation might provide an effective approach to limit AD pathology in people at-risk through cardiovascular risk factors.

### Interaction With AD Factors

Hypertension is often co-morbid with other metabolic risk factors such as diabetes, obesity and dyslipidemia, with <20% of all cases occurring in isolation (83). It is thought that this grouping can be attributed to an insulin resistance syndrome promoted by obesity and the closely related metabolic cardiovascular syndrome (84). Approximately 30% of coronary events in men and 70% in women have been attributed to clusters of two or more cardiovascular risk factors with hypertension being only one component of a complex interplay of risk factors (83). Risk factors for coronary complications are also associated with AD, and therefore, it is likely that the higher the number of risk factors for coronary events is, the higher the risk is for AD. For example, smoking and hypertension when comorbid with T2DM confer a higher risk for AD relative to individuals with T2DM only (85). Thus, the clustered risk profile of an individual with hypertension makes them more susceptible to AD through shared risk factors.

The modifying role of APOE genotype in the relationship between hypertension and AD has been studied by Kester et al. (86) in a patient population. It was found that the link between hypertension and levels of CSF tau and p-tau 181 was modulated by APOE genotype but differed between individuals depending on the characteristics of the genotype present. Homozygous APOE ε4 carriers with hypertension had significantly higher levels of CSF tau and ptau-181 compared to individuals of the same genotype, but no hypertension. Furthermore, APOE ε4 genotype did not interact with the relationship between hypertension and Aβ42, suggesting that tau pathology alone is directly modified by genotype. Data from the Seattle Longitudinal study spanning over a 21-year period revealed that hypertension synergises with the effects of APOE ε4 on the rate of cognitive decline (87). These findings demonstrate the potential benefit of combining hypertension and APOE genotype factors in identifying high risk for cognitive decline.

### Effects of Treatment

Antihypertensive treatment has shown promising results in lowering risk of future cognitive decline. In a 3-year community cohort study, subjects taking antihypertensive medication (primarily diuretics) had a lower incidence of dementia compared to untreated controls (88). The use of other antihypertensive medication (calcium antagonists or β-blockers) only showed a reduction in AD risk in a subpopulation with pre-treatment systolic blood pressure >160 mm Hg or diastolic blood pressure >95 mm Hg. Furthermore, untreated subjects with dementia had twice the rate of cognitive decline compared to dementia patients receiving antihypertensive therapy. A retrospective national cohort study on antihypertensive drug use and AD risk in diabetic individuals demonstrated that the most effective treatment for lowering AD risk is seen with angiotensin II receptor blocker use (24% lower risk of dementia), followed by diuretics (14%), angiotensin-converting-enzyme inhibitors (11%) and b-blockers (4%) (79). More recently, a meta-analysis of prospective cohort studies revealed that individuals with treated hypertension had a reduced risk for developing dementia compared to those not taking medication (89). Overall, there is consistent evidence that points to the benefits of antihypertensive therapy as both primary (i.e., reducing risk of new dementia diagnoses in hypertensive patients) and secondary prevention of AD (i.e., reducing rate of cognitive decline in established dementia).

## Chronic Kidney Disease

### Epidemiology

Chronic kidney disease (CKD) is characterized by impaired kidney function as defined by glomerular filtration rate (GFR) of <60 mL/min per 1.73 m^2^, or signs of kidney damage of 3 months or longer duration. Two of the major causes of CKD are diabetes and hypertension with diabetes accounting for 30–50% of all CKD cases (90). Based on a survey of non-institutionalized adults in the USA, it was estimated that hypertension is present in 23% of adults without CKD, 36% in those with stage 1 CKD, 48% in stage 2, 60% in those with stage 3 CKD and 84% in those with stages 4 and 5 CKD (91). The relationship appears to be bi-directional as kidney function is a critical mechanism for regulating blood pressure and therefore CKD typically results in further deterioration of blood pressure control (92).

Individuals with chronic kidney disease have been found to have an elevated risk for cognitive decline. Etgen et al. (93) carried out a systematic meta-analysis to investigate the relationship between CKD and cognitive decline. Their analysis included over 54,000 participants and concluded that individuals with CKD are more likely to experience cognitive decline than those without CKD. Findings from the Chronic Renal Insufficiency Cohort Cognitive Study (94) showed that individuals with advanced CKD were more likely to show clinically significant cognitive impairment in most cognitive domains compared to those with mild to moderate CKD. Furthermore, a meta-analysis including 54,779 individuals showed that for each 10 mL decline in the GFR value below the clinical threshold for impairment (60 mL/min per 1.73 m^2^), the risk for cognitive decline increases by 11% (95). A study of 1,015 postmenopausal women with diagnosed coronary heart disease reported a 15–25% increase in risk of cognitive decline per 10 ml/min/1.73 m^2^ decrement n eGFR measured (96). Similarly, findings from the Rush Memory and Aging Project reported an association between CKD and dementia where global cognitive decline was comparable to 3 years of aging for each eGFR reduction of 15 ml/min/1.73 m^2^ (97). These studies suggest a linear relationship between loss of kidney function and subsequent cognitive decline.

### Mechanism

Despite strong evidence pointing to the association between CKD and cognitive decline, the mechanisms involved in the interaction remain unclear with several interacting mechanisms having been proposed. Firstly, the failure to eliminate metabolic waste products through kidney failure adversely impacts multiple organs, including the brain. It has been proposed that of the many uremic toxins normally excreted through the kidneys, uric acid, parathyroid hormone (PTH) and indoxyl sulfate are most likely to contribute to the development of cognitive decline in individuals with CKD (10). Urinary toxins such as PTH can accumulate and pass through the blood-brain barrier. Elevated PTH levels have been shown to associate with to hyperparathyroidism, which can in turn impair cognitive performance (98, 99).

Vascular injury is a key clinical characteristic of CKD which has been hypothesized to accelerate AD progression (100). Similar to the mechanism proposed in hypertension, the reduced vascular reactivity and permeability seen in CKD may initiate or accelerate the core AD pathophysiological process (101). A relationship between albuminuria, a marker of microvascular dysfunction in both kidney and brain tissue, and AD has been demonstrated by Oh et al. (102), thus arguing that as both organs are low resistance end organs, they are particularly prone to injury through high blood flow.

The renin-angiotensin system (RAS) activation plays a key role in the development of CKD (103) and has also been found to be linked to AD progression. Continuous RAS activation in rodent models results in a reduction in cognitive functioning which in turn is linked to a reduction in cerebral surface blood flow and higher levels of oxidative stress (104).

### Interaction With AD Factors

APOE ε4 is a major risk factor for AD, but surprisingly has been found to slow disease progression in CKD. While APOE ε2 genotype has been associated with lowered glomerular filtration rate and CKD, the ε4 allele provides protection against CKD progression (105). To investigate this further Chu et al. (106) examined the role of these two APOE alleles in CKD progression in a prospective cohort. There is consistent evidence linking APOE ε4 carriership to decreased risk of CKD, although the relationship appears to be strongest in Caucasians (107). The exact mechanisms for this unexpected interaction remain unclear.

The risk of AD is known to increase with age and such effects also contribute to AD risk in individuals with CKD. Cheng et al. (108) carried out a cohort study and found that AD risk in CKD patients was similar for both men and women, however, the age specific relative risk was the highest for the youngest group and lowest for the elderly. Taken together these findings highlight the importance of dementia monitoring of CKD to identify cognitive decline in younger individuals as well as those without genetic risk.

### Effects of Treatment

The various health implications of CKD have led researchers to investigate whether CKD treatment could slow down the detrimental effects it has on brain functioning. Various studies have looked at the effects of renal replacement therapies on Aβ clearance. Peritoneal dialysis, a treatment for CKD, has been found to reduce Aβ plasma levels in both CKD patients and APP/PS1 mice model associated with early-onset AD suggesting Aβ clearance might be a promising therapeutic strategy to prevent the accumulation of amyloid plaques (109). Lower levels of serum Aβ in CKD patients receiving dialysis have been confirmed in another study (110) which the authors interpreted as evidence for protective action of dialysis through peripheral Aβ clearance. This interpretation is however potentially controversial, as reduction of Aβ in CSF and blood is also a hallmark of amyloid being deposited in cortical amyloid plaques. Renal transplantation, a treatment for end-stage kidney disease has also been shown to reduce the likelihood of developing dementia. For instance, a successful renal transplantation has been shown to significantly improve cognitive and psychomotor performance on measures of processing speed, attention and executive functioning (111). Improvements in cognitive performance have also been demonstrated in dialysed patients after kidney transplantation with the degree of cognitive improvement linked to factors such as duration of CKD, age and renal function post-surgery (112).

## Prevention Strategy Based on Comorbidities

The added risk that comorbidities pose on healthy cognitive aging has received substantial attention from researchers, however, the application of this knowledge into active prevention strategies has been held back by underinvestment and difficulties in stratifying risk at scale. Monitoring populations based on high-risk comorbidities for AD and controlling their risk factors but also fast-tracking individuals into disease-modifying treatments as they become available could limit the burden of AD significantly.

### Control of Risk Factors

The modifiable risk factors that deserve the highest level of attention are mostly linked to either cardiovascular and metabolic risk factors such as diabetes, hypertension and chronic kidney disease, and lifestyle factors such as smoking, physical activity, diet, mental and social stimulation. The evidence for the impact of interventions aimed at reducing modifiable risk factors on dementia risk is accumulating thus paving the way for prevention programmes. The Finnish Geriatric Intervention Study to Prevent Cognitive Impairment and Disability (113) was carried out to investigate the effects of lifestyle intervention on slowing cognitive decline in individuals with cognitive abilities at mean levels or lower than expected for their age according to Finnish population norms. They found that a multi-domain intervention including diet, exercise, cognitive training and control of vascular risk factors can improve or maintain cognitive abilities in individuals aged 60–77. It has been suggested that such findings indicate the possibility of lowering dementia risk in individuals with cardiovascular risk factors through control of their underlying cardiovascular disease (114). Similarly, Santos et al. (115) carried out a multidisciplinary rehabilitation program to study the effects of cognitive rehabilitation, computer assisted cognitive training, speech therapy, occupation therapy, art therapy, physical training and cognitive stimulation on cognitive decline. They found that patients with mild AD in the intervention arm experienced improvements in cognition, quality of life and a reduction in depressive symptoms. No improvement was seen in individuals with moderate AD, supporting the idea that risk reduction interventions hold the best chances of success if implemented in the earliest stages of AD.

### Disease Modification Therapies

Excess accumulation and deposition of Aβ and intracellular neurofibrillary tangles composed of tau protein are at the heart of AD pathology, and therefore, have led to numerous secondary disease prevention strategies aiming to target these key components. The cortical Aβ burden is determined by the balance between its production and clearance, and an offset in this balance can lead to the accumulation of Aβ as seen in AD (116). Aβ is synthesized from amyloid precursor protein by γ-secretase, and thus, the blocking of these enzymes has been a major focus of drug development. However, it is now understood that Aβ clearance and degradation rather than synthesis are more critical in the accumulation of Aβ, and therefore, pharmacological targets have shifted focus (117). Despite the efforts, therapies targeted against Aβ clearance have been unsuccessful until recently. Aducanumab, a human monoclonal antibody that selectively targets Aβ build-ups has been shown to decrease the levels of aggregated Aβ in early-stage AD in a dose dependent fashion with an associated marginal effect on cognition and functional ability (118). After its successful approval with the FDA in the US, it is likely that aducanumab will follow similar trajectories in other regions including Europe.

Early tau targeting therapies have primarily focussed on either inhibition of kinases, tau aggregation or stabilization of microtubules, however, trials have been mostly unsuccessful due to toxicity and lack of efficacy (119). The majority of tau targeted therapies currently in clinical trials are immunotherapies. For instance, a phase 2 study began in January 2021 for passive immunization with JNJ-63733657, which has so far been found to eliminate pathogenic tau seeds in cell-based assays and to inhibit the spread of tau pathology in a mouse model. It has been argued that tau is likely to be a better target than Aβ once cognitive impairment is detectable, because it correlates better with clinical symptoms than Aβ accumulation (119).

## Monitoring of At-Risk Groups

### Monitoring Blood-Based Biomarkers

In a healthcare system of limited resources, a key challenge to either treatment strategy is the effective identification of preclinical dementia risk and the assessment of the efficacy of potentially toxic therapies in otherwise cognitively healthy or minimally impaired individuals.

Rapid developments in the methodology of blood-based assays now represent a viable option for the task of monitoring risk and assessing treatment effects in early-stage AD. Blood-based testing is significantly less expensive and less invasive than both CSF (requires lumbar puncture) and PET (requires administration of radioactive ligands). It also represents a routine clinical procedure that does not require specialist skills or equipment. Blood sampling is already used to screen large populations for other conditions due to its availability in a variety of settings from clinics to home based testing. Accumulating research demonstrates the effectiveness of use of blood-based biomarkers in distinguishing individuals with biological AD from controls across disease stages (120). The Amyloid/Tau/Neurodegeneration (ATN) network has been proposed as a framework for the assessment of the biological trajectory of AD irrespective of clinical symptoms (121). While the ATN network groups biomarkers from imaging to biofluids, the ease of testing and low cost of blood-based biomarkers, make them an ideal candidate for assessing biological change in large cohorts. In terms of amyloid, until recently its low concentrations in blood and the high affinity of albumin to it meant that well-established assays such as the enzyme-linked immunosorbent assay (ELISA) did not offer a reliable method for its detection. However, novel methods such as immunomagnetic reduction IMR have been shown to consistently separate AD patients from controls (120). In a recent study, a high-precision plasma β-amyloid 42/40 ratio assay (immunoprecipitation and liquid chromatography-mass spectrometry), accurately predicted amyloid cortical burden with an area under the curve (AUC) of 0.88. The performance was further improved when age and APOE4 carriership were added to the model (AUC 0.94) (122). Tau biomarker blood assays have also seen dramatic improvement, especially through the single molecule arrays (SiMOA) technology. In AD CSF p-tau181 has been shown to be abnormal even before Aβ markers (CSF or PET) reach abnormal levels, with elevated levels correlating with cortical Aβ pathology (123). Although both t-tau and p-tau are elevated in CSF levels in individuals with AD pathology, t-tau is non-specific as it is increased in any condition involving neural injury e.g., stroke, traumatic brain injury (124, 125). In contrast, p-tau change appears to be specific to AD pathology (126, 127). Encouragingly, ptau-181 and 217 in blood-based assays also demonstrate utility in differentiating between AD and non-AD pathology, thus offering an option for differential diagnosis at the earliest stages of disease. Neurofilament light (NfL) is a structural protein released through any demyelinating process. Its levels in CSF have been shown to be consistent AD neurodegeneration biomarkers and can be used effectively to distinguish individuals with AD from controls (128). More recently, plasma NfL concentration has also been demonstrated to differentiate AD from non-demented controls and to have a strong significant correlation with CSF NfL (129). Taken together, the blood biomarker assays currently available offer an opportunity to detect the biological process underlying AD (120). In addition, p-tau plasma assays appear to have a role in the differential diagnosis of AD from other dementia causing pathologies (130) while NFL has already been shown to be a dynamic marker of neurodegeneration which can be used to assess potential disease modification effects (131).

### Monitoring Cognition

Digital technologies offer an until now unavailable opportunity to evaluate cognition remotely without a supervised clinician. Such novel digital technologies provide cost-effective, sensitive, objective and multidimensional alternatives to pen and paper cognitive tests that can be used at scale and at high density. They offer the definition of intra-individual trajectories in healthy aging which then can detect the subtle deviations in cognitive functioning part of the earliest disease manifestations.

#### Passive Monitoring by Wearable Devices

In addition to active cognitive monitoring, digital and wearable devices offer the option to derive cognitive information through gathering data on users' behavioral patterns and ability to interact with the devices. These technologies are widespread and do not require specialized equipment, offer immediate access to information, increase sensitivity, put an extremely low burden on the healthcare system and offer a unique approach to map cognition to biological changes in real time (132). Passive monitoring devices can collect rich high-frequency longitudinal data that is objective, inexpensive and is of low burden to the patient. Below we list some of the main areas of development in this field.

##### Monitoring Gait and Physical Activity

Wearable devices can track early signs of gait impairment, which can start up to 12 years before any cognitive impairment can be detected (133). By measuring gait speed, step variability, rhythm, asymmetry and postural control it has been possible to detect early signs of AD (134) and associations with AD biomarkers (135). The relevance of gait to AD detection lies in its complexity. It is a cognitive task that requires function from multiple domains such as attention, executive function, visuospatial function and motor processing for successful task execution (133). Wearables can also provide objective measures of physical activity levels through accelerometry which in turn allows the reconstruction of diurnal variation in activity. This offers an additional option for risk detection as specific patterns of physical activity and sleep associate with dementia risk (136, 137). The feasibility of gathering accelerometry data at scale has been demonstrated through studies such as the recording of data from 100 k UK Biobank participants (138).

##### Monitoring Fine Motor Movements

In addition to gait and physical activity, smart devices can also gather rich and valuable information about cognitive and motor abilities through the user's engagement with the device. These data on fine motor control, language abilities and processing speed, can be used to build predictive models for early disease detection. For example, Ntracha et al. (139) used touchscreen typing characteristics (participants were asked to type stories on the phone) to build a model with diagnostic ability that distinguishes MCI patients from controls. The best performing model had accuracy of 80% (AUC = 0.75), which is in a similar range to many other dementia prediction models that use large cohort data (140, 141).

#### Active Monitoring by Digital Devices

##### Monitoring Using Cognitive Tests

Prodromal AD can be detected by tests focusing on cognitive functions supported by structures affected first by the pathophysiological process e.g., temporal memory for bound features as a proxy of hippocampal function (142, 143). The sensitivity of this method can be improved further by high density longitudinal testing that generates an individual's expected trajectory which can be used to detect the subtle deviations that occur in early AD. Digital technologies have attempted to combine these two approaches. For example, the Mezurio app used a paired associate smartphone-based test of inanimate vs. animate objects presented visually (thought to reflect perirhinal function) to evaluate episodic memory at periods of up to 11 days of mid-life individuals at increased risk for AD. It found that longer term retention periods (5 days or longer) are needed to reliably uncover subtle cognitive deficits in people at risk for AD (144). The task also highlighted the value of short, frequent and flexible cognitive tests in terms of acceptability for the individual (145).

##### Monitoring Using Eye-Tracking Technologies

The oculomotor system is gaining increasing attention as a potential biomarker for dementia in AD. Individuals with MCI show deficits in executive functions e.g., inhibitory control (146). Eye-movement error-correction tasks (antisaccade tasks) can measure this in a simple paradigm and have been shown to detect early signs of AD before standard neuropsychological tests do (147). Novel digital technologies are emerging that combine eye-tracking technologies with cognitive tests and offer a multi-dimensional approach to detecting cognitive impairment. Data from built-in laptop web cameras show a strong correlation with high frame rate eye trackers on measures of visual memory (148), and therefore, have the potential to screen individuals without specialized laboratory equipment. A recent study has confirmed that eye-tracking can also be applied to smartphone cameras (149), providing an even more accessible tool. All in all, the rapid developments in digital monitoring are providing a broad gamut of options for cost-effective monitoring of individuals at risk at the comfort of their home.

## Discussion

The failure of AD treatment strategies implemented in its clinical stages has shifted the focus of secondary prevention strategies to its preclinical stages (15). Improved understanding of the risk factors for AD has opened an opportunity to identify individuals at high risk for developing AD from mid-life (150). These individuals can then be monitored up using scalable technology to allow the identification of preclinical dementia and the subsequent implementation of prevention strategies, be it via addressing of risk factors or disease modification therapies as they become available. Developments in blood-based AD biomarkers are increasingly recognized as a mature option for scalable and low-cost alternatives to previous invasive diagnostic methods. Similarly, digital biomarkers offer the opportunity to monitor functional and behavioral changes in individuals through passive and active monitoring that lower the burden to the healthcare system and put the risk monitoring to patient's own hands.

In this paper, we focused on selecting cardiovascular diseases as the chief strategy for the identification of at-risk group. This is based on the strong evidence detailing the link between such comorbidities and the development of cognitive decline and dementia. The rationale is further strengthened by studies showing that targeted interventions of these risk factors can result in better dementia outcomes, especially when implemented early. In addition, conditions such as hypertension, diabetes and CKD tend to be looked after through close supervision by either primary and/or secondary care which makes them highly accessible for secondary prevention.

We propose that the mechanics of creating an at-risk cohort should be based on models estimating the risk of developing dementia in the next 5–10 years by combining demographic, genetic and cardiovascular severity data. We have previously shown that detecting Alzheimer's disease pathology among cognitively normal individuals can be achieved using models incorporating age, sex and APOE4 carriership (AUC 0.82) (155) and that this can be increased further (AUC 0.84), for example by adding body mass index, a proxy for insulin resistance (151). Individuals deemed to be at high risk can then be longitudinally (e.g., annually) monitored via plasma biomarkers for signs of AD pathophysiology being triggered (e.g., change in ptau-181 levels). In addition, digital biomarkers can be used to establish a cognitive baseline which through regular testing (e.g., 6 monthly) can detect subtle deviations in ability. Wearable technology can provide information on activity and sleep which in turn can further improve the risk prediction models. Taken together, such monitoring programmes can then focus efforts to those that would most benefit from targeted multi-domain interventions of specific risk factors (113), but also create the infrastructure for identifying individuals with specific AD pathology who would then be candidates for disease modification treatment using e.g., amyloid clearance therapies. The lack of such AD biomarker-informed research and healthcare infrastructure has been a major barrier to the development of novel dementia research therapies (152).

The creation of risk-based preclinical AD cohorts for the purposes of secondary prevention is not without its limitations. Firstly, the monitoring of risk would, in most cases, result in disclosure of information relating to personal and familial risk of a currently untreatable condition. The creation of such infrastructure therefore will require carefully designed risk disclosure protocols. Even so, it may be that a proportion of individuals choose not to engage with a programme that may result in risk disclosure which can limit the programme's impact. In addition, as shown in other screening programmes, the scale required will mean that the risk exists for psychological distress through false positives. Equally, there is the possibility that early disease cases may be missed through test inaccuracies or system errors, as demonstrated by a failure of primary care to follow-up abnormal screening mammograms in 27–73% of cases within 6 months (153). An AD secondary prevention programme may suffer an even exacerbated form of the problem due to the huge number of people who may fit the criteria for being at-risk. The disclosure of risk to a large population of people would need to be met with the availability of trained clinicians who can interpret, communicate the results and, arrange for follow-up through appropriately resourced clinical services. Secondly, large scale collection and linkage of sensitive health data places an emphasis on security and data privacy infrastructure that can withstand the ongoing global challenges in cybersecurity. Thirdly, data collection is likely to take place in collaboration with third parties that develop and maintain the digital technologies. This raises the issue of data ownership and intellectual property in regards to healthcare. A fourth factor is one of appropriate consent. Awareness of the implications of risk disclosure, data security and data ownership as well as other issues as they arise are going to be key to establishing ability for individuals to provide informed consent. This is likely to be a major challenge for any healthcare system and one that would be liable to legal risks in individuals deemed to be at risk of cognitive impairment.

In summary, improved understanding of the risk factors for AD combined with novel scalable diagnostic methods provide an until now unavailable opportunity for the secondary prevention of the chief cause of death in the developed world. Our success would depend on the ability of healthcare decision makers to invest in the required infrastructure and on the reformulation of dementia and cognitive health in public discourse: from an untreatable condition that is part of aging to a brain health state which can be modified by the right intervention at the right time for the right person.

## Author Contributions

TO and IK drafted and reviewed the manuscript. Both authors contributed to the article and approved the submitted version.

## Funding

IK has current funding through grants from the Medical Research Council (Dementias Platform UK grant number MR/L023784/2), National Institute of Health Research (Development and Skills Enhancement Award grant number NIHR301616), and the NIHR Oxford Health Biomedical Research Centre.

## Conflict of Interest

TO was employed by Sharp Therapeutics. IK was a medical advisor to Sharp Therapeutics.

## Publisher's Note

All claims expressed in this article are solely those of the authors and do not necessarily represent those of their affiliated organizations, or those of the publisher, the editors and the reviewers. Any product that may be evaluated in this article, or claim that may be made by its manufacturer, is not guaranteed or endorsed by the publisher.
